# PSMA-RECAP: [^177^Lu]Lu-PSMA-617 retreatment in metastatic castration-resistant prostate cancer

**DOI:** 10.3389/fonc.2026.1831393

**Published:** 2026-08-28

**Authors:** Gokce Belge Bilgin, Cem Bilgin, Ann T. Packard, Brian J. Burkett, Elif Su Oztekin, Matthew P. Thorpe, Daniel S. Childs, Miguel Muniz, Ronen Stoff, Yalda Nikanpour, Jacob J. Orme, Eugene D. Kwon, Geoffrey B. Johnson, Oliver Sartor, Derek R. Johnson, Ayse Tuba Kendi

**Affiliations:** 1 Department of Radiology, Mayo Clinic, Rochester, MN, United States; 2 Department of Oncology, Mayo Clinic, Rochester, MN, United States; 3 Department of Immunology, Mayo Clinic, Rochester, MN, United States; 4 Department of Urology, Mayo Clinic, Rochester, MN, United States; 5 Transformational Prostate Cancer Research Center, East Jefferson Hospital Cancer Center, Metairie, LA, United States

**Keywords:** Lu 177 PSMA, mCRPC, pluvicto, radioligand therapy (RLT), theranosctics

## Abstract

**Purpose:**

Radioligand therapy (RLT) with [^177^Lu] Lu-PSMA-617 has shown significant benefits in metastatic castration-resistant prostate cancer (mCRPC), including prolonged overall survival (OS) and progression-free survival (PFS), as well as improved quality of life. For patients previously treated with [^177^Lu]Lu-PSMA-617 who subsequently experience disease progression, retreatment with [^177^Lu] Lu-PSMA-617 has emerged as a potential approach, yet data on the outcomes of RLT retreatment remains limited. This study aimed to assess the efficacy and safety of retreatment RLT in mCRPC.

**Methods:**

Clinical and imaging data of patients who underwent at least two cycles of [^177^Lu] Lu-PSMA-617, followed by a second course of treatment with [^177^Lu] Lu-PSMA-617 between March 2018 and December 2024, were retrospectively reviewed. Endpoints included biochemical response, imaging response, toxicity, PSA-PFS, rPFS, and OS. Radiologic and biochemical responses were evaluated using RECIST 1.1 criteria and PSA levels, respectively.

**Results:**

A total of 589 patients underwent [^177^Lu] Lu-PSMA-617 RLT, of whom 20 (3.4%, 20/589) received RLT retreatment. The median age was 71.3 years (range: 63.7–95.2), and the median follow-up was 73.3 months (95% CI, 35.7–77.2 months) after the initial treatment and 21.7 months (95% CI, 16.9–26.0 months) after retreatment. The initial treatment involved a median of 4.5 cycles (range: 2–6). Following the first course of RLT, 15 patients (75%, 15/20) had exhibited a >50% decline in PSA levels, including 9 who achieved a >90% decrease. Additionally, 12 patients (60%, 12/20) had demonstrated a complete or partial imaging response, while 3 (15%, 3/20) patients had stable disease, and 5 (25%, 5/20) patients had progressive radiologic disease. Patients received a median of 4 retreatment cycles (range, 1–6), and the median interval between initial RLT and retreatment RLT was 33.1 months (IQR, 16.2–48.9 months; range, 10.7–58.8 months). A PSA decline of ≥50% was observed in 9 out of 20 patients (45%, 9/20). An imaging response was observed in 10 patients (50%, 10/20), with 4 achieving a complete response and 6 achieving a partial response, whereas 10 patients (50%, 10/20) showed progression. The median PSA-PFS was 9.8 months (95% CI, 4.1–17.9 months), and the median rPFS was 8.2 months (95% CI, 3.3–17.9 months). Eight deaths occurred during follow-up. The estimated Kaplan Meier OS rates were 83.6% at 12 months (95% CI, 57.3%–94.4%) and 53.7% at 24 months (95% CI, 28.3%–73.7%). Grade 2 renal toxicity was observed in one patient (5%, 1/20). Three patients (15%, 3/20) developed Grade 2 anemia. Notably, no grade 3 or grade 4 adverse events were observed during either treatment period.

**Conclusion:**

In our cohort, retreatment with [¹^77^Lu]Lu-PSMA-617 RLT was associated with biochemical and imaging responses in some patients, with observed survival outcomes that support further investigation. However, given the retrospective single-arm design and small sample size, large prospective studies are needed to more rigorously evaluate the safety, efficacy, and optimal patient selection for retreatment RLT.

## Introduction

Metastatic castration-resistant prostate cancer (mCRPC) represents a clinically challenging disease, defined by progression despite androgen deprivation therapy (1). While prognosis remains poor, advances over the past decade have markedly improved patient outcomes, with multiple life-prolonging therapies now integrated into standard care (2).

Following the pivotal phase III VISION trial, [^177^Lu]Lu-PSMA-617 radioligand therapy (RLT) received FDA approval in 2022 and has since been established as a standard treatment option for patients with PSMA-positive mCRPC previously exposed to androgen receptor pathway inhibitors (ARPIs) and taxane-based chemotherapy (3). In March 2025, the indication for [^177^Lu]Lu-PSMA-617 was broadened following the phase III PSMAfore trial, allowing its administration in patients with mCRPC who had received prior ARPI therapy and were candidates for deferral of chemotherapy, thereby facilitating its earlier incorporation into the treatment paradigm (4).

Although many patients derive significant clinical benefit from RLT, disease progression is common, and subsequent treatment options remain limited. Given its favorable toxicity profile and proven survival benefit, there is growing interest in retreatment with [^177^Lu]Lu-PSMA-617 in selected patients who demonstrate ongoing or recurrent PSMA expression (5–11). However, in the current literature, dedicated data evaluating the efficacy and safety of retreatment RLT remain scarce. In this study, we present real-world outcomes from a single-institution cohort undergoing retreatment with [^177^Lu]Lu-PSMA-617.

## Methods

### Study design and patient selection

This is a single-center, retrospective analysis conducted in accordance with the Declaration of Helsinki and approved by the Institutional Review Board. Clinical records and imaging data were reviewed for patients with histologically confirmed mCRPC who received at least two cycles of initial [^177^Lu]Lu-PSMA-617 RLT, followed by a second course of [^177^Lu]Lu-PSMA-617 between March 2018 and December 2024. Patients were followed until May 2025. Patients without longitudinal follow-up or those who did not provide research authorization were excluded. Demographic and clinical characteristics were extracted from electronic medical records.

Adverse events were evaluated using laboratory data and clinical documentation at baseline, before each treatment cycle, and at 3- and 6-month follow-up visits after retreatment initiation, when available. Laboratory parameters assessing kidney, liver, and bone marrow function were documented. Toxicities were graded according to the Common Terminology Criteria for Adverse Events, version 5.0 (CTCAE v5.0) (12).

For patients with measurable soft-tissue lesions, responses to the initial and retreatment RLT courses were evaluated independently according to the Response Evaluation Criteria in Solid Tumors (RECIST), version 1.1. For each course, imaging obtained at treatment completion was compared with the baseline imaging obtained before the start of that respective RLT course (13). Outcomes were categorized as complete response, partial response, stable disease, or progressive disease. Biochemical response to [^177^Lu]Lu-PSMA-617 rechallenge therapy was evaluated using serial serum prostate-specific antigen (PSA) measurements. PSA response was assessed according to Prostate Cancer Working Group 3 (PCWG3) criteria and the EANM/SNMMI procedure guideline (14, 15).

### Radiopharmaceutical administration of RLT

All patients underwent a pre-treatment [^68^Ga] Ga-PSMA-11 PET/CT or an [^18^F] DFCPyL PET/CT scan to identify and evaluate PSMA-avid lesions, which was defined as those with an uptake greater than that observed in the liver by visual assessment (15). Patients were considered candidates for RLT if the molecular imaging PSMA expression score was 2 or greater (3, 15, 16). The patients’ medical histories, recent imaging data, clinical conditions, and laboratory tests obtained one day before the planned treatment date were evaluated. The treatment regimen has been previously described (17).

### Outcomes and statistical analysis

The primary study endpoints were biochemical response, imaging response, and toxicity. Secondary endpoints included PSA progression-free survival (PSA-PFS), radiologic progression-free survival (rPFS), and overall survival (OS). Overall survival (OS) was calculated from the first cycle of retreatment RLT to the date of death or last follow-up. PSA-PFS was defined as the time from the first cycle of retreatment RLT to PSA progression, and rPFS as the time from the first cycle of retreatment RLT to radiologic progression.

## Results

A total of 589 patients underwent [177Lu] Lu-PSMA-617 RLT, of whom 20 (3.4%, 20/589) received RLT retreatment. The median age was 71.3 years (range: 63.7–95.2), and the median follow-up was 73.3 months (95% CI, 35.7–77.2 months) after the initial [177Lu] Lu-PSMA-617 RLT and 21.7 months (95% CI, 16.9–26.0 months) after retreatment. The detailed patient characteristics are presented in **Table 1**. Other treatments received before initial RLT and intervening therapies administered between initial RLT and retreatment RLT are listed in **Supplementary Material 1**.

**Table 1:** Patient characteristics.

Patient characteristics	Value
Age*	71.3 (63.7–95.2)
Gleason score*	8 (7–10)
ECOG - no. of patients (n, %)	
*0 or 1*	19 (95%, 19/20)
≥ 2	1 (5%, 1/20)
Sites of metastases (n, %)	
*Bone*	17 (85%, 17/20)
*Lymph node*	19 (95%, 19/20)
*Liver*	1 (5%, 1/20)
*Lung*	3 (15%, 3/20)
*CNS*	1 (5%, 1/20)
Number of bone lesions (n, %)	
*1-4*	4 (23%, 4/17)
*5-10*	6 (35%, 6/17)
*>10*	7 (42%, 7/17)
Previous systemic therapies (n, %)	
*ADT*	20 (100%, 20/20)
*Chemotherapy*	20 (100%, 20/20)
*ARPI*	18 (90%, 18/20)
*Immunotherapy*	1 (5%, 1/20)
*^[223Ra]^Radium*	1 (5%, 1/20)
Initial RLT	
*Number of cycles, median (range)*	4.5 cycles (2–6)
*^177^Lu activity, median (range) in [GBq]*	
Per cycle	7.4 GBq (4-8.2)
Cumulative administered activity	37 GBq (11-44.4)
Retreatment RLT	
*Number of cycles, median (range)*	4 cycles (1–6)
^177^Lu activity, median (range) in [GBq]	
Per cycle	7.4 GBq (7.4-7.4)
Total cumulative administered activity across initial and retreatment courses	48.2 GBq (29.6-88.8)

**Abbreviations:** ADT, Androgen Deprivation Therapy; ARPI, Androgen Receptor Pathway Inhibitor; CNS, central nervous system; GBq, Giga-becquerel; RLT, radioligand therapy.

* Median (range).

**Figure 1:**
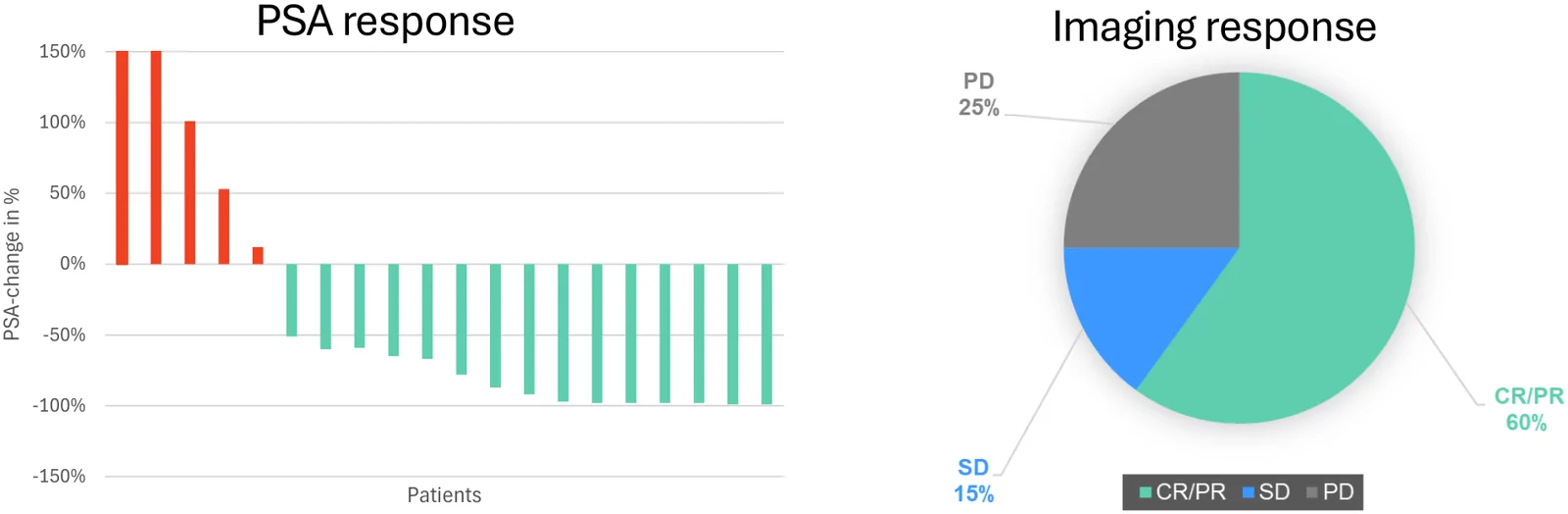
Biochemical and radiologic treatment responses to initial [^177^ Lu]Lu-PSMA-617 RLT.

**Figure 2:**
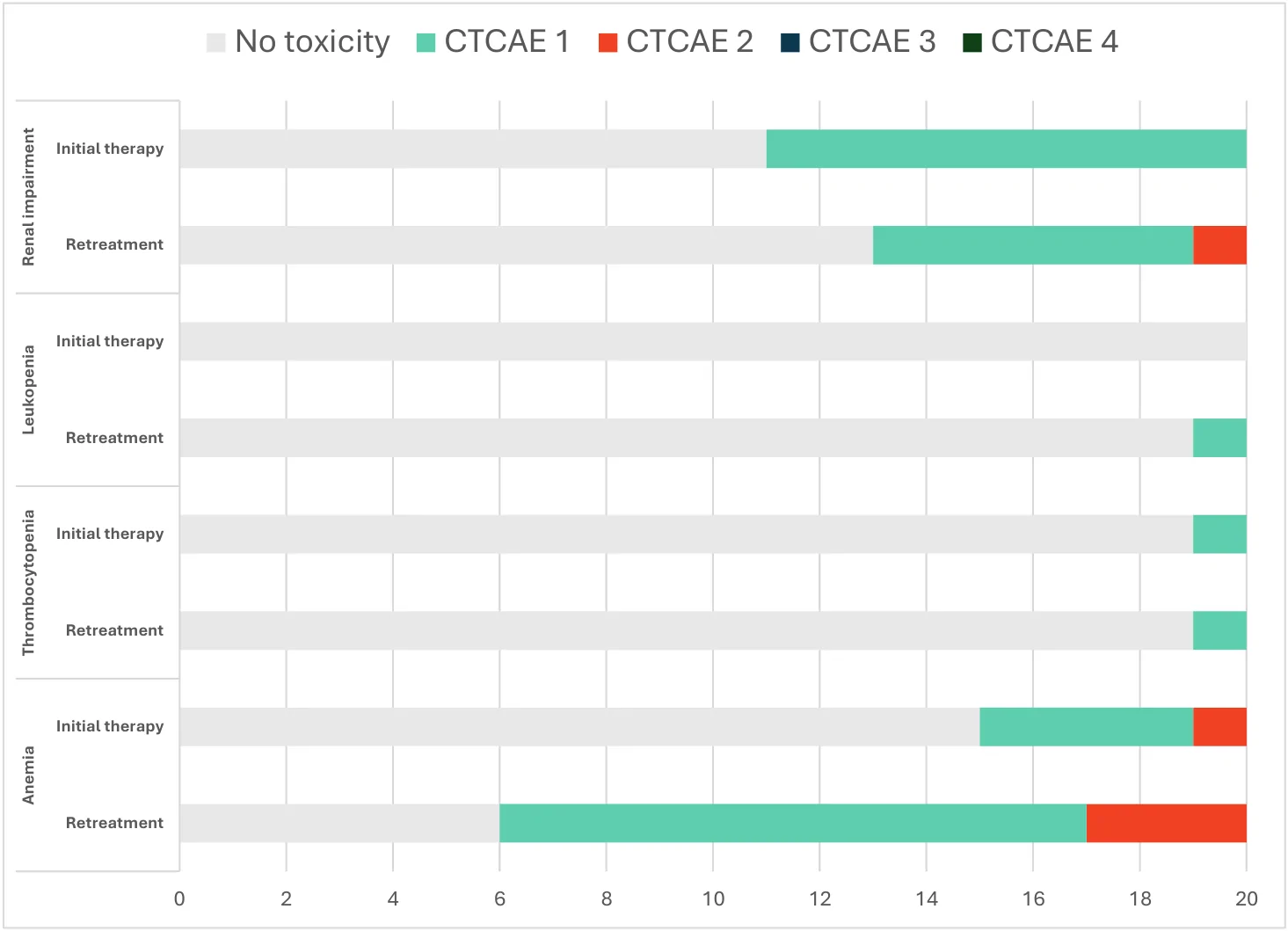
Hematologic and renal toxicities graded according to the common terminology criteria for adverse events (СТСАЕ).

The initial treatment comprised a median of 4.5 cycles (range: 2–6). Following the first course of RLT, fifteen patients (75%, 15/20) experienced a >50% decline in PSA levels, including nine (45%, 9/20) who achieved a >90% reduction. Twelve patients (60%, 12/20) demonstrated a positive imaging response, with seven (35%, 7/20) achieving complete response and five (25%, 5/20) partial response. Three patients (15%, 3/20) had stable disease, and five (25%, 5/20) exhibited overall radiologic progression while showing interval improvement or stability in select lesions (**Figure 1**). Grade 2 anemia occurred in one patient (5%, 1/20). No Grade 3–4 hematologic toxicities or Grade 2–4 renal impairment were observed during or after the initial course of RLT. **Figure 2** provides a detailed overview of toxicity noted following initial and retreatment RLT.

**Figure 3:**
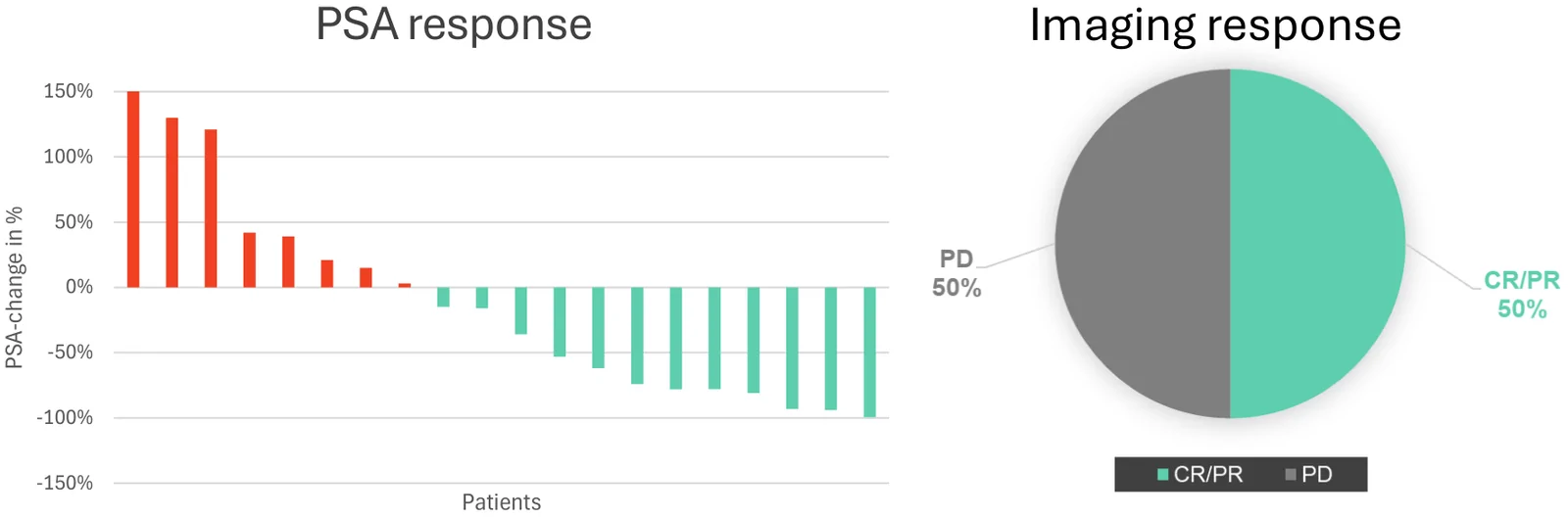
Biochemical and radiologic treatment responses to retreatment [1^77^ Lu]Lu-PSMA-617 RLT.

**Figure 4:**
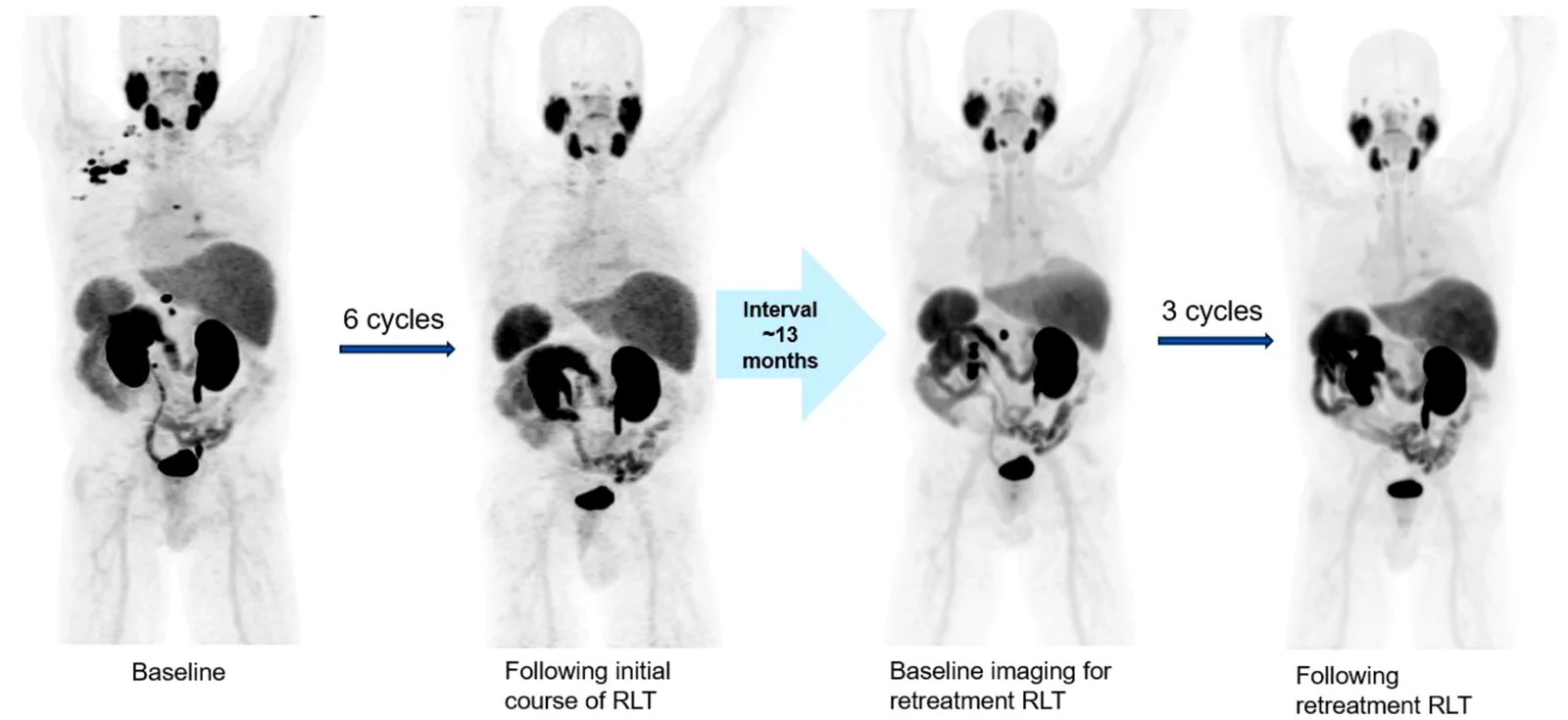
Representative case showing excellent response to six initial cycles of [^177^Lu]Lu-PSMA-617 RLT, progression after 13 months, and complete response following three retreatment cycles, sustained at 6 months.

Patients received a median of 4 retreatment cycles (range, 1–6), and the median interval between initial RLT and retreatment RLT was 33.1 months (IQR, 16.2–48.9 months; range, 10.7–58.8 months). A ≥50% decline in PSA levels was achieved in nine patients (45%, 9/20), including three (15%, 3/20) with a >90% reduction. Among these responders, seven maintained their biochemical response at 3 months and six at 6 months. A positive imaging response was observed in ten patients (50%, 10/20), comprising four (20%, 4/20) complete and six (30%, 6/20) partial responses, whereas the remaining ten patients (50%, 10/20) exhibited radiologic disease progression (**Figures 3**, **4**). One patient developed Grade 2 (5%, 1/20) renal impairment after retreatment RLT. Hematologic toxicities included Grade 2 anemia in three patients (15%, 3/20). No Grade 3 or 4 toxicities were observed during treatment or throughout the follow-up period.

**Figure 5:**
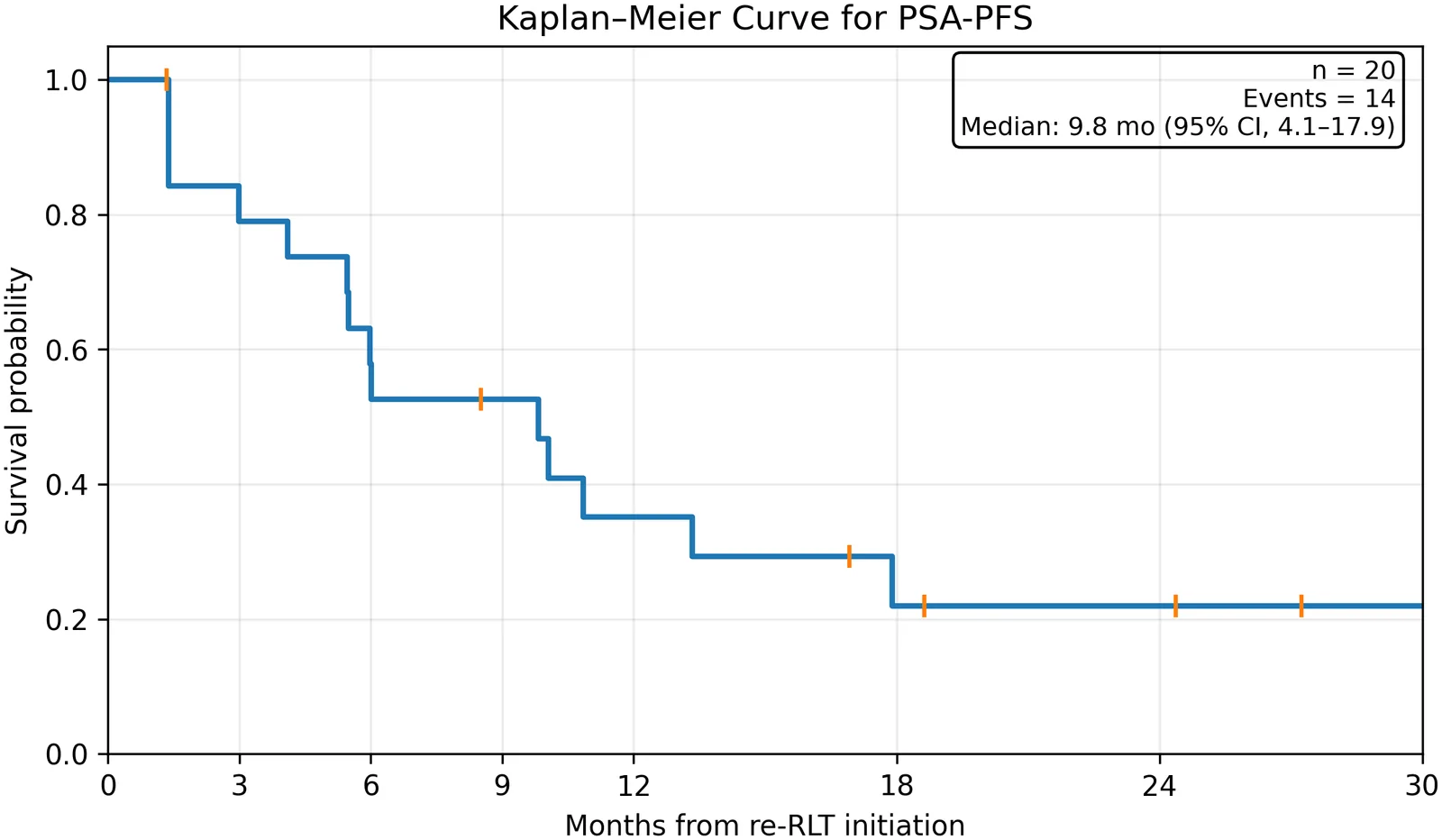
Kaplan–meier curve of PSA-progression free survival following retreatment with [¹^77^Lu]Lu-PSMA-617 radioligand therapy.

**Figure 6:**
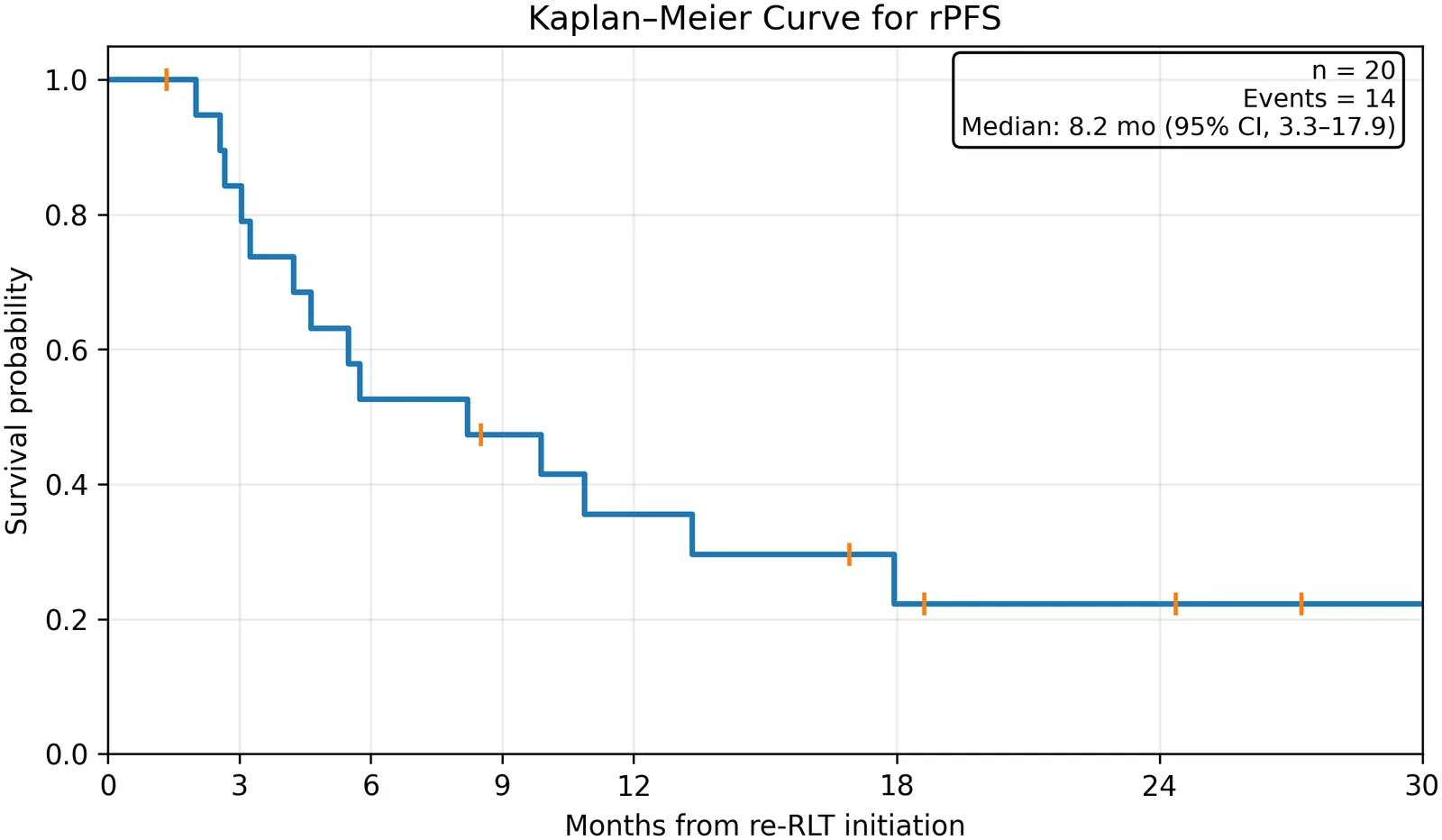
Kaplan–meier curve of radiologic-progression free survival following retreatment with [¹^77^Lu]Lu-PSMA-617 radioligand therapy.

**Figure 7:**
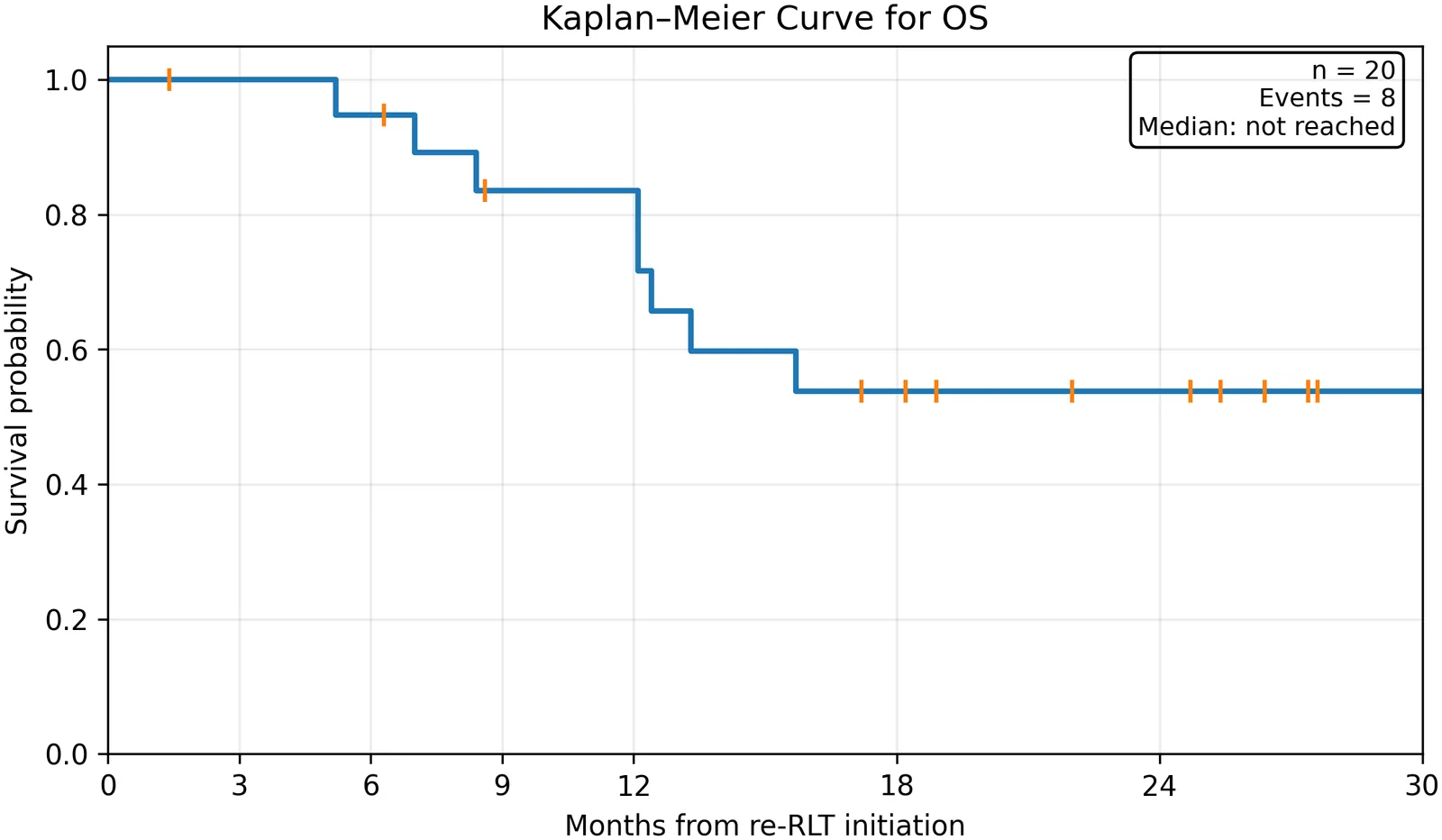
Kaplan–meier curve of overall survival following retreatment with [¹^77^Lu]Lu-PSMA-617 radioligand therapy.

The median PSA-PFS was 9.8 months (95% CI, 4.1–17.9 months) (**Figure 5**), and the median rPFS was 8.2 months (95% CI, 3.3–17.9 months) (**Figure 6**). Eight deaths occurred during follow-up. Median OS was not reached during the observed follow-up period because the Kaplan-Meier survival curve did not cross 50%. The estimated Kaplan-Meier OS rates were 83.6% at 12 months (95% CI, 57.3%–94.4%) and 53.7% at 24 months (95% CI, 28.3%–73.7%) (**Figure 7**).

## Discussion

Our study evaluating the efficacy and safety of [^177^Lu]Lu-PSMA-617 retreatment yielded several important observations. First, nearly half of the cohort exhibited a biochemical and radiologic response. Second, only a small number of Grade 2 toxicities were observed, and no Grade 3–4 events were reported. Third, biochemical responses appeared to be maintained in most PSA responders at 3- and 6-month follow-up. These findings are important because they suggest that retreatment RLT may warrant further investigation as a potential therapeutic approach for patients with mCRPC who have previously received an initial course of RLT.

In our cohort of 20 patients undergoing retreatment with [177Lu]Lu-PSMA-617 RLT, 45% achieved a ≥50% decline in PSA levels, and 50% demonstrated a partial or complete radiologic response. These outcomes align with findings from prior studies evaluating RLT retreatment. Santo et al. evaluated 18 patients who underwent retreatment RLT and reported a ≥50% PSA decline in 44% of patients, with 56% showing either complete or partial response or stable disease (9). In a larger cohort, Rosar et al. reported that 57.4% of patients (27/47) achieved a ≥50% PSA decline following up to three rechallenge series, with therapeutic benefit maintained across as many as 17 cumulative treatment cycles (8). Notably, our study also included five patients who exhibited a heterogeneous radiologic response to initial RLT, characterized by the coexistence of responding lesions alongside progressing or newly emerging sites. Of these, two achieved partial response following retreatment RLT. These findings may suggest that retreatment with [^177^Lu]Lu-PSMA-617 might represent a feasible strategy for selected patients with sustained PSMA expression, although the therapeutic efficacy of subsequent courses may be less robust than that achieved with the initial treatment.

Toxicity data in the current literature vary considerably across studies. In this cohort, the most common toxicities following retreatment RLT were hematologic, with Grade 2 anemia observed in 15% and Grade 2 renal impairment in 5% of patients. No Grade 3 or Grade 4 toxicities were observed during follow-up period. Rosar et al. reported that 10.6% of patients experienced Grade 3 or 4 adverse events across up to three rechallenge RLT cycles, predominantly hematologic, with one case of renal toxicity (8). Also, they noted a slight increase in the frequency of Grade 1 and 2 events during successive treatment series. Yordanova et al. observed Grade 3 toxicity in 27% of patients, again primarily hematologic, with one patient developing Grade 3 renal impairment (11). Although uncommon, Grade 4 toxicities have been documented in the literature. Santo et al. reported three cases of Grade 4 adverse events during post-retreatment follow-up, including thrombocytopenia, renal failure, and pancytopenia with concurrent renal failure (9). However, it is important to note that patients with mCRPC are often heavily pretreated, and their bone marrow reserve and renal function may already be compromised before initiation of retreatment RLT. Furthermore, the absence of control arms in existing rechallenge studies limits the ability to attribute long-term toxicities exclusively to [^177^Lu]Lu-PSMA-617, as such effects may reflect the cumulative burden of prior systemic therapies rather than the isolated impact of RLT.

As [^177^Lu]Lu-PSMA-617 RLT receives broader regulatory approval with its recent FDA label expansion, an increasing number of patients with mCRPC are expected to undergo RLT earlier in their disease course and may subsequently become candidates for retreatment. This development may lead to important clinical questions regarding the patient selection, safety, efficacy, and optimal timing of retreatment RLT. Currently, no established guidelines exist to inform the extended use of [^177^Lu]Lu-PSMA-617 RLT in terms of efficacy and safety. Prospective trials in the United States and Europe, including RE-LuPSMA (NCT06288113) (18) and ReaLuP (NCT06866938) (19), are currently underway to evaluate clinical outcomes associated with [^177^Lu]Lu-PSMA-617 retreatment. In addition, multicenter registries and prospective clinical trials are currently investigating the safety and efficacy of [^225^Ac]Ac-PSMA-617, both as monotherapy and in combination with [^177^Lu]Lu-PSMA-617 in tandem approaches, among patients who have progressed following conventional [^177^Lu]Lu-PSMA-617 RLT (20, 21). Furthermore, the phase II NET-RETREAT trial (NCT05773274) (22) is investigating [^177^Lu]Lu-DOTATATE retreatment versus everolimus in patients with NETs, contributing further data on RLT retreatment strategies.

Our study has several limitations. First, this was a retrospective, single-arm analysis without a control or comparator group; therefore, the observed outcomes after retreatment RLT should be interpreted as associations rather than evidence of causal benefit compared with alternative management strategies or no further treatment. Although all patients who underwent retreatment RLT during the study period were included, retreatment was not administered according to a prespecified retreatment protocol. Instead, treatment decisions were made at the discretion of the treating physicians based on clinical status, prior treatment response, imaging findings, laboratory parameters, available therapeutic alternatives, and patient preference. Second, the small sample size of 20 patients limits statistical power and generalizability, particularly given the clinical heterogeneity of the cohort, including variability in the number of retreatment cycles, the interval between initial RLT and retreatment RLT, and intervening or subsequent systemic and local therapies. Therefore, formal subgroup analyses, including stratification by response to initial RLT, treatment interval, disease burden, or intervening therapies, were not performed because they would be underpowered and at high risk of overinterpretation. Third, survival data should be interpreted cautiously, as these estimates is conditional on patients having survived long enough and remained clinically eligible to receive retreatment RLT, introducing potential survivor/immortal-time bias and limiting comparability with pivotal trials of initial RLT. Finally, because PSA assessment, imaging follow-up, and treatment decisions were performed as part of routine clinical care rather than according to a standardized prospective protocol, the timing of progression and toxicity assessment may have varied across patients. Larger prospective studies with standardized retreatment criteria, follow-up schedules, and comparator groups are needed to better define durability of response, late toxicity, and the patients most likely to benefit from retreatment RLT.

## Conclusion

In this retrospective single-arm cohort, retreatment RLT after an initial course of RLT was associated with observed PSA responses and disease-control outcomes in a subset of patients with mCRPC. However, given the small sample size, absence of a comparator group, heterogeneity in prior and subsequent therapies, and potential selection and survivor biases, these findings should be interpreted as exploratory and hypothesis-generating. Larger prospective studies with standardized retreatment criteria and appropriate comparator groups are needed to determine the clinical benefit of retreatment RLT and to identify patients most likely to benefit.

## Data Availability

The original contributions presented in the study are included in the article/**Supplementary Material**. Further inquiries can be directed to the corresponding author. The raw data supporting the conclusions of this article will be made available by the corresponding author upon reasonable request.

## Supplementary material

Supplementary Table 1 Prior therapy before initial RLT.

Supplementary Table 2 Intervening therapy between initial RLT and re-RLT.
