# Supplementary material for: PSMA-RECAP: [177Lu]Lu-PSMA-617 retreatment in metastatic castration-resistant prostate cancer
Source: Front Oncol. 2026 Aug 28;16:1831393. doi: 10.3389/fonc.2026.1831393 (PMC13561789; doi:10.3389/fonc.2026.1831393)
Supplement: Supplementary file 1 [file Table1.docx]

**Supplemental Data for Prior Treatments**

| **Prior therapy before initial RLT** | **n / 20** | **%** |
| --- | --- | --- |
| Abiraterone | 19 | 95% |
| Docetaxel | 17 | 85% |
| Bicalutamide | 12 | 60% |
| Enzalutamide | 10 | 50% |
| Cabazitaxel | 8 | 40% |
| Carboplatin | 8 | 40% |
| Darolutamide | 6 | 30% |
| PARP inhibitor | 4 | 20% |
| Apalutamide | 2 | 10% |
| Pembrolizumab | 2 | 10% |
| Ra-223 | 2 | 10% |
| Sipuleucel-T | 2 | 10% |
| Other systemic therapy | 2 | 10% |

| **Intervening therapy between initial RLT and re-RLT** | **n / 20** | **%** |
| --- | --- | --- |
| Any documented non-ADT intervening therapy | 13 | 65% |
| Radiotherapy / SBRT / IMRT | 8 | 40% |
| Any ARPI | 8 | 40% |
| Enzalutamide | 4 | 20% |
| Darolutamide | 3 | 15% |
| Abiraterone | 2 | 10% |
| Bicalutamide | 1 | 5% |
| Any taxane chemotherapy | 4 | 20% |
| Docetaxel / Taxotere | 2 | 10% |
| Cabazitaxel | 2 | 10% |
| Carboplatin/Cisplatin chemotherapy | 2 | 10% |
| PARP inhibitor / olaparib | 1 | 5% |
